# Joint association of depression and physical activity with incident cardiometabolic multimorbidity (CMM) among middle-aged and older adults in China

**DOI:** 10.1038/s41598-026-50014-2

**Published:** 2026-04-21

**Authors:** Zhaoxuan Liu, Rongyu Qi, Lifeng Qu, Guili Wang, Jiyu Li, Xiwen Liu

**Affiliations:** 1 Department of Vascular Surgery, Central Hospital Affiliated to Shandong First Medical University, No.105, Jiefang Road, Lixia District, Jinan, 250013 China; 2 Center for Disinfection Guidance, Jinan Center for Disease Control and Prevention, No. 988, Biandian East Road, Huaiyin District, Jinan, 250013 China; 3 Department of Electrocardiogram, Jinan Hospital, Lixia District, Jinan, 250013 China

**Keywords:** Depression, VPA, Cardiometabolic multimorbidity, Cardiology, Diseases, Endocrinology, Health care, Medical research, Risk factors

## Abstract

Depression and physical inactivity are both associated with cardiometabolic disorders; however, their joint effects on the development of CMM, defined as the coexistence of ≥ 2 cardiometabolic conditions, remain unclear. We aimed to investigate the combined association of depression and vigorous physical activity (VPA) with incident CMM and to examine whether this association differed across metabolic status. This prospective cohort study included 2,661 adults aged ≥ 45 years without CMM at baseline (2011) from the China Health and Retirement Longitudinal Study. Participants were followed through 2020 (median follow-up: 9 years), during which 797 incident CMM cases were identified. Depression and VPA were jointly categorized into four groups. Cox proportional hazards models with multiple imputation were used to estimate hazard ratios (HRs) and 95% confidence intervals (CIs). Estimated glucose disposal rate (eGDR) was evaluated as a potential effect modifier using stratified analyses and interaction tests. Sensitivity analyses were performed using alternative metabolic indicators, including the triglyceride–glucose (TyG) index, waist-to-height ratio (WHtR), and cardiometabolic index (CMI). Compared with participants without depression who engaged in VPA (reference), those with both depression and no VPA had the highest risk of CMM (HR 1.93, 95% CI 1.52–2.44), followed by those with depression but engaging in VPA (HR 1.84, 95% CI 1.41–2.39) and those without depression but no VPA (HR 1.52, 95% CI 1.22–1.89). The associations were consistent across metabolic strata defined by eGDR. No evidence of interaction was observed between depression and VPA (P for interaction > 0.05). A dose–response relationship was observed across joint exposure categories (P for trend < 0.001). Sensitivity analyses yielded similar results. Depression and lack of VPA jointly increase the risk of CMM in middle-aged and older adults. Engagement in VPA may partially mitigate the elevated cardiometabolic risk associated with depression. These findings highlight the importance of integrated mental health and lifestyle interventions for preventing CMM.

**Supplementary Information:**

The online version contains supplementary material available at 10.1038/s41598-026-50014-2.

## Introduction

CMM, defined as the coexistence of two or more cardiometabolic conditions, has emerged as a major global health challenge^1^. Recent large-scale epidemiological studies have shown that the prevalence of CMM is increasing substantially, particularly among middle-aged and older adults^2,3^, and is associated with markedly elevated risks of mortality and disability^4^. For example, evidence from population-based cohorts such as the UK Biobank and global burden studies indicates that individuals with CMM have a two- to four-fold higher risk of adverse health outcomes compared with those without multimorbidity^5,6^.

Depression is one of the most prevalent mental health disorders among middle-aged and older adults and has been consistently linked to an increased risk of cardiometabolic diseases^7,8^. Previous epidemiological studies have reported associations between depression and hypertension, type 2 diabetes, coronary heart disease, and stroke^9–12^. These studies have largely examined these conditions as isolated outcomes. However, limited evidence exists regarding the role of depression in the development of CMM as a clustered outcome. From a pathophysiological perspective, depression may contribute to cardiometabolic dysfunction through multiple mechanisms, including dysregulation of the hypothalamic–pituitary–adrenal axis^13^, chronic low-grade inflammation^14,15^, insulin resistance^16^, and adverse health behaviors^17^, all of which are common pathways underlying cardiometabolic diseases.

Physical activity is a well-established protective factor against cardiometabolic diseases and premature mortality^18^. Regular physical activity improves blood pressure, glucose metabolism, and lipid profiles and is also recognized as an effective non-pharmacological intervention for depression^19^. VPA, in particular, may confer greater cardiometabolic benefits compared with lower-intensity activity^20,21^. Importantly, depression and physical activity are closely interrelated: individuals with depression are more likely to engage in insufficient physical activity, while physical inactivity may exacerbate depressive symptoms and accelerate metabolic deterioration^22^. Despite this bidirectional relationship, few studies have examined the joint association of depression and physical activity with CMM, particularly using prospective data from middle-aged and older populations.

Insulin resistance is considered a central mechanism linking multiple cardiometabolic conditions. The eGDR is a simple surrogate marker of insulin sensitivity that has been increasingly applied in cardiometabolic and cardiovascular epidemiological studies^23^. Importantly, it remains unclear whether the joint effects of depression and physical activity on CMM vary across metabolic status. Furthermore, validating findings using alternative metabolic indicators, such as the TyG index, WHtR, and cardiometabolic index (CMI), may improve the robustness of conclusions and reduce potential bias associated with a single marker. Therefore, using data from the China Health and Retirement Longitudinal Study (CHARLS), we conducted a prospective cohort study to investigate the joint association of depression and physical activity with the risk of incident CMM among middle-aged and older adults. Specifically, we aimed to: (1) evaluate the combined effects of depression and physical activity on CMM risk; (2) assess whether these associations were consistent across different levels of metabolic status, stratified by eGDR; and (3) examine the robustness of the findings through sensitivity analyses using alternative metabolic indicators. This study seeks to provide evidence to support integrated mental health and lifestyle interventions for the prevention of CMM in aging populations.

## Methods

### Study design and population

**Figure 1:**
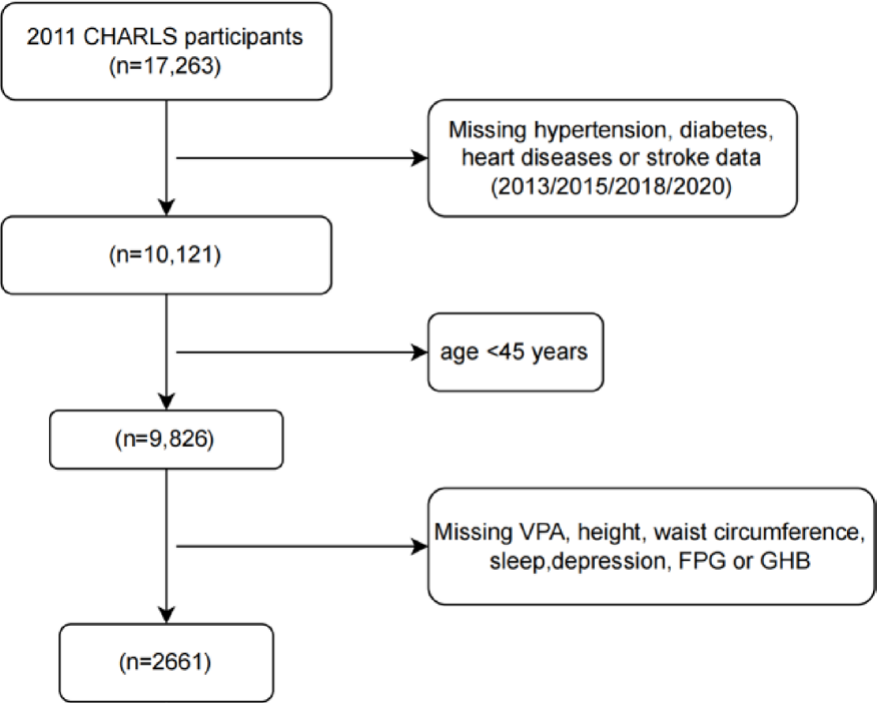
Flowchart of participant selection.

This study was based on data from the China Health and Retirement Longitudinal Study (CHARLS), a nationally representative longitudinal survey of Chinese adults aged 45 years and older that employed a multistage, stratified probability sampling design. Data were collected through face-to-face interviews, physical examinations, and laboratory tests, as described previously. The flowchart of participant selection is shown in Fig. 1.

For the present analysis, the 2011 wave was used as baseline. Participants free of CMM at baseline were followed up in 2013, 2015, 2018, and 2020 to identify incident CMM. Participants without follow-up information were excluded.

### Definition of CMM

CMM was defined as the coexistence of two or more of the following physician-diagnosed conditions: hypertension, diabetes mellitus, cardiovascular disease (including coronary heart disease), and stroke, which was consistent with previous epidemiological studies^24^. Incident CMM was identified when a participant was first diagnosed with at least two of these conditions during follow-up. The time-to-event variable was calculated as the interval between baseline (2011) and the follow-up wave at which CMM was first identified. Participants who did not develop CMM during follow-up were censored at their last follow-up.

### Assessment of depression

Depressive symptoms were assessed at baseline using the 10-item Center for Epidemiologic Studies Depression Scale (CESD-10). This assessment comprises ten items, which include: “Bothered by things,” “Had trouble keeping mind,” “Felt depressed,” “Felt everything I did was an effort,” “Felt fearful,” “Sleep was restless,” “Felt lonely,” “Could not get going,” “Felt hopeful about the future,” and “Happy.” Responses to each item are rated on a scale of 0 (rarely or never), 1 (sometimes), 2 (most of the time), or 3 (always). It is important to note that the items “Felt hopeful about the future” and “Happy” are scored in reverse. The cumulative score can range from 0 to 30. Research suggests that a cutoff score of 10 points demonstrates adequate sensitivity and specificity; thus, we classify a CESD10 score of ≥ 10 points as indicative of depression^25,26^. Depression was assessed at baseline only and assumed to represent long-term exposure.

### Assessment of VPA

VPA was assessed using a self-reported questionnaire that asked participants about the frequency of activities requiring substantial physical exertion during a typical week. Vigorous activities were defined as those that caused rapid breathing or shortness of breath, such as carrying heavy loads, digging or farming, aerobic exercise, fast cycling, or cycling while carrying heavy goods. Participants were instructed to recall only activities lasting at least 10 min per session. Information on VPA was collected, and participants were categorized according to their overall level of physical activity based on established criteria. For the main analyses, VPA was selected as the primary exposure due to its stronger association with cardiometabolic health outcomes compared with lower-intensity physical activity^27–29^.

### Joint exposure of depression and physical activity

To examine the combined effects of depression and VPA, a joint exposure variable was constructed with four categories:

1. No depression + VPA
2. Depression + VPA
3. No depression + No VPA
4. Depression + No VPA

The group of participants without depression who were VPA served as the reference category in all analyses. Depression and VPA were assessed at baseline and were assumed to reflect relatively stable exposure patterns over time, although potential changes during follow-up could not be captured.

### Metabolic indicators

The eGDR was used as the primary metabolic indicator and calculated based on established equations incorporating waist circumference, hypertension status, and glycated hemoglobin. Stratified Cox regression analyses were performed according to tertiles of eGDR to assess whether the associations differed across metabolic subgroups. For sensitivity analyses, three alternative metabolic indicators were examined: the TyG index, WHtR, and CMI. These indicators were included separately in adjusted models to evaluate the robustness of the associations.

### Covariates

Baseline covariates were selected a priori based on previous literature and included age^30^, gender^31^, marital status^32^, educational level^30^, smoking status^32,33^, alcohol consumption^32^, kidney disease^34^, respiratory function^31^, sleep duration^35^, social activity participation^36^, instrumental activities of daily living (IADL) and history of falls^37^. All covariates were measured at baseline. Sleep duration was categorized into three groups: < 7 h (coded as 1), 7–8 h (coded as 2), and > 8 h (coded as 3). Social activity participation was assessed using 11 activity items available in the CHARLS database, including interacting with friends, playing ma-jong/cards/chess, providing help to others living apart, attending clubs, participating in community organizations, engaging in voluntary or charity work, caring for a sick or disabled adult living apart, attending educational or training courses, stock investment, using the internet, and other activities. For each activity, participation was coded as 1 (yes) or 0 (no), and a composite social activity score was calculated by summing the number of activities, with higher scores indicating greater social engagement. Instrumental activities of daily living (IADL) were used to assess functional status. The CHARLS questionnaire includes six items: eating, dressing, transferring, toileting, bathing, and grooming. Each item was scored as 0 (“no difficulty”) or 1 (“having difficulty but able to perform,” “requiring assistance,” or “unable to perform”). The total IADL score was obtained by summing across items, with higher scores indicating poorer functional status. Educational attainment was classified into four levels: below primary school, primary school, middle school, and above middle school, corresponding to scores of 1, 2, 3, and 4, respectively.

### Missing data and multiple imputation

To handle missing baseline covariate data, a combined approach of case exclusion and multiple imputation was employed. Individuals with extensive missing data on key variables were excluded from the analysis. For the remaining missing values, multiple imputation by chained equations (MICE) was performed under the missing-at-random (MAR) assumption. The imputation model included all variables used in the primary analyses and the outcome indicator. Five imputed datasets were generated, and the estimates from each were combined using Rubin’s rules to obtain pooled hazard ratios (HRs) with their corresponding 95% confidence intervals (CIs).

### Statistical analysis

Baseline characteristics were summarized as means with standard deviations for continuous variables and as frequencies with percentages for categorical variables. Differences across groups were assessed using appropriate statistical tests. Cox proportional hazards regression models were used to estimate the associations of depression, VPA, and their joint exposure with the risk of incident CMM. Two sequential models were constructed: Model 1 was adjusted for age and sex; Model 2 was further adjusted for marital status, education level, instrumental activities of daily living (IADL), smoking status, alcohol consumption, sleep duration, social activity, history of falls, and other relevant covariates. Depression and VPA were assessed at baseline and were assumed to reflect relatively stable exposure patterns over time, although potential changes during follow-up could not be captured. To account for baseline metabolic differences, stratified Cox models were performed according to tertiles of eGDR. This approach allowed the baseline hazard to vary across metabolic strata while assessing the association between joint exposure and CMM risk. To evaluate potential effect modification, interaction terms between the joint exposure (depression and VPA) and eGDR categories were included in the Cox models. Joint exposure models were constructed by categorizing participants into four groups based on depression status and VPA, with “No Depression + VPA” as the reference group. Trend tests were performed by modeling this joint exposure variable as an ordinal variable to assess dose–response relationships. Sensitivity analyses were conducted by additionally adjusting for alternative metabolic indicators, including the TyG index, WHtR, and cardiometabolic index (CMI), in separate models to evaluate the robustness of the findings. Subgroup analyses stratified by age and sex were performed, and formal interaction tests were conducted to assess heterogeneity across subgroups. The proportional hazards assumption was assessed using Schoenfeld residuals. To address potential reverse causality, additional analyses were conducted by excluding participants who developed CMM during the early follow-up period (2013). Cox proportional hazards models were then refitted using the same covariate adjustments as in the primary analyses. Both individual exposure models (depression and VPA) and joint exposure models were re-estimated to assess the robustness of the associations. All statistical analyses were conducted using R software (R Foundation for Statistical Computing, Vienna, Austria; version 4.5.0; https://www.r-project.org/), with a two-sided *p* value < 0.05 considered statistically relevant.

## Results

### Baseline characteristics

A total of 2661 participants free of CMM at baseline were included in the analysis. Participants with depression were more likely to be older, physically inactive, and have poorer functional status compared with those without depression. Individuals with Non-VPA tended to have higher prevalence of unfavorable metabolic profiles and lifestyle risk factors. Baseline characteristics stratified by depression and physical activity status are presented in Table 1.

**Table 1:** Baseline characteristics of study participants according to depression and VPA.

	No Depression + VPA	Depression + VPA	No Depression + Non-VPA	Depression + Non-VPA	*p*
N	676	377	1019	589	
Age mean ± SD	56.36 (7.70)	57.56 (7.36)	58.67 (8.80)	60.27 (9.08)	< 0.001
Gender					< 0.001
Male	388 (57.4%)	153 (40.6%)	436 (42.8%)	162 (27.5%)	
Female	288 (42.6%)	224 (59.4%)	583 (57.2%)	427 (72.5%)	
Education					< 0.001
1	301 (44.5%)	226 (59.9%)	422 (41.4%)	335 (56.9%)	
2	156 (23.1%)	82 (21.8%)	206 (20.2%)	125 (21.2%)	
3	154 (22.8%)	55 (14.6%)	260 (25.5%)	85 (14.4%)	
4	65 (9.6%)	14 (3.7%)	131 (12.9%)	44 (7.5%)	
Married					< 0.001
YES	636 (94.1%)	341 (90.5%)	928 (91.1%)	501 (85.1%)	
NO	40 (5.9%)	36 (9.5%)	91 (8.9%)	88 (14.9%)	
BMI mean ± SD	23.22 (3.44)	22.85 (3.84)	24.16 (3.69)	23.79 (4.15)	< 0.001
Hypertension					< 0.001
YES	121 (17.9%)	89 (23.6%)	255 (25.0%)	207 (35.1%)	
NO	555 (82.1%)	288 (76.4%)	764 (75.0%)	382 (64.9%)	
Diabetes					< 0.001
YES	16 (2.4%)	19 (5.0%)	57 (5.6%)	47 (8.0%)	
NO	660 (97.6%)	358 (95.0%)	962 (94.4%)	542 (92.0%)	
Lung disease					< 0.001
YES	40 (5.9%)	52 (13.8%)	70 (6.9%)	63 (10.7%)	
NO	636 (94.1%)	325 (86.2%)	949 (93.1%)	526 (89.3%)	
Heart disease					< 0.001
YES	28 (4.1%)	44 (11.7%)	110 (10.8%)	110 (18.7%)	
NO	648 (95.9%)	333 (88.3%)	909 (89.2%)	479 (81.3%)	
Stroke					0.002
YES	6 (0.9%)	9 (2.4%)	14 (1.4%)	21 (3.6%)	
NO	670 (99.1%)	368 (97.6%)	1005 (98.6%)	568 (96.4%)	
Kidney disease					0.008
YES	32 (4.7%)	29 (7.7%)	40 (3.9%)	41 (7.0%)	
NO	644 (95.3%)	348 (92.3%)	979 (96.1%)	548 (93.0%)	
Height mean ± SD	1.59 (0.08)	1.56 (0.08)	1.58 (0.08)	1.56 (0.08)	< 0.001
Weight mean ± SD	59.13 (10.49)	55.92 (10.56)	60.70 (11.39)	57.73 (11.53)	< 0.001
Waist mean ± SD	82.76 (12.39)	82.18 (12.04)	85.69 (12.52)	85.59 (12.33)	< 0.001
Respiratory function mean ± SD	334.49 (131.87)	272.88 (110.45)	300.96 (122.29)	251.04 (102.51)	< 0.001
LDL mean ± SD	114.12 (33.17)	116.56 (32.56)	118.93 (35.47)	116.41 (34.72)	0.044
FPG mean ± SD	105.69 (22.34)	106.70 (30.97)	108.85 (32.39)	109.06 (35.38)	0.118
TG mean ± SD	121.53 (123.43)	122.29 (97.25)	136.79 (99.38)	142.12 (113.09)	0.001
GHB mean ± SD	5.16 (0.62)	5.27 (0.88)	5.25 (0.73)	5.31 (0.92)	0.007
IADL					< 0.001
0	601 (88.9%)	264 (70.0%)	903 (88.6%)	385 (65.4%)	
1	51 (7.5%)	55 (14.6%)	65 (6.4%)	75 (12.7%)	
2	16 (2.4%)	34 (9.0%)	27 (2.6%)	48 (8.1%)	
3	4 (0.6%)	15 (4.0%)	16 (1.6%)	41 (7.0%)	
4	4 (0.6%)	7 (1.9%)	3 (0.3%)	27 (4.6%)	
5	0 (0.0)	2 (0.5%)	5 (0.5%)	13 (2.2%)	
Smoke					< 0.001
YES	307 (45.4%)	134 (35.5%)	339 (33.3%)	153 (26.0%)	
NO	369 (54.6%)	243 (64.5%)	680 (66.7%)	436 (74.0%)	
Alcohol					< 0.001
YES	317 (46.9%)	146 (38.7%)	352 (34.5%)	170 (28.9%)	
NO	359 (53.1%)	231 (61.3%)	667 (65.5%)	419 (71.1%)	
VPA	676 (100%)	377 (100%)	0 (0.0)	0 (0.0)	< 0.001
Sleep time					< 0.001
1	275 (40.7%)	243 (64.5%)	418 (41.0%)	379 (64.3%)	
2	350 (51.8%)	113 (30.0%)	505 (49.6%)	173 (29.4%)	
3	51 (7.5%)	21 (5.6%)	96 (9.4%)	37 (6.3%)	
Social activity					0.002
0	330 (48.8%)	203 (53.8%)	428 (42.0%)	317 (53.8%)	
1	226 (33.4%)	130 (34.5%)	408 (40.0%)	186 (31.6%)	
2	86 (12.7%)	38 (10.1%)	133 (13.1%)	71 (12.1%)	
3	28 (4.1%)	4 (1.1%)	36 (3.5%)	11 (1.9%)	
4	5 (0.7%)	1 (0.3%)	7 (0.7%)	1 (0.2%)	
5	0 (0.0)	1 (0.3%)	4 (0.4%)	2 (0.3%)	
6	1 (0.1%)	0 (0.0)	2 (0.2%)	1 (0.2%)	
7	0 (0.0)	0 (0.0)	1 (0.1%)	0 (0.0)	
History of falls					< 0.001
YES	80 (11.8%)	103 (27.3%)	110 (10.8%)	141 (23.9%)	
NO	596 (88.2%)	274 (72.7%)	909 (89.2%)	448 (76.1%)	
Depression	0 (0.0)	377 (100%)	0 (0.0)	589 (100%)	< 0.001
eGDR mean ± SD	12.84 (1.88)	12.69 (1.92)	12.33 (2.04)	11.99 (2.18)	< 0.001
TyG mean ± SD	8.56 (0.63)	8.59 (0.64)	8.72 (0.64)	8.73 (0.71)	< 0.001
CMI mean ± SD.	1.60 (4.62)	1.44 (1.92)	1.93 (2.78)	2.08 (3.38)	0.006
WHtR mean ± SD	0.52 (0.08)	0.53 (0.08)	0.54 (0.08)	0.55 (0.08)	< 0.001

### Incidence of CMM

During a median follow-up of 9 years, 797 participants developed CMM. The cumulative incidence of CMM increased progressively across joint exposure categories, from 17.5% in participants without depression who engaged in VPA to 42.6% in those with both depression and no VPA.

### Association of depression and VPA with CMM

In multivariable Cox proportional hazards regression analyses, depression was independently associated with a higher risk of incident CMM. VPA was inversely associated with the risk of incident CMM. These associations remained statistically relevant after adjustment for sociodemographic, behavioral, and health-related covariates (Table 2).

**Table 2:** Associations of depression and VPA with incident CMM.

	Value	HR	p.value	95% CI
Model 1	Age	1.037	< 0.001	1.030–1.045
	Gender	0.825	0.008	0.715–0.951
Model 2	Age	1.034	< 0.001	1.025–1.043
	Gender	0.853	0.172	0.678–1.072
	Education	1.088	0.035	1.006–1.176
	Married	1.109	0.382	0.879–1.398
	IADL	1.143	< 0.001	1.071–1.22
	Smoke	1.011	0.919	0.822–1.243
	Alcohol	1.019	0.832	0.855–1.214
	VPA	0.654	< 0.001	0.558–0.766
	Kidney disease	1.637	< 0.001	1.26–2.127
	Respiratory function	1	0.16	1–1.001
	Sleep time	0.995	0.924	0.888–1.114
	Social activity	1.04	0.343	0.959–1.126
	History of falls	1.115	0.237	0.931–1.336
	Depression	1.481	< 0.001	1.268–1.729

The proportional hazards assumption was assessed using Schoenfeld residuals. No violations were observed (global test *P* = 0.183), although borderline results were noted for depression (*P* = 0.056) and VPA (*P* = 0.074). Cumulative incidence was considered as a marker of metabolic status and evaluated as a potential effect modifier. Stratified Cox regression analyses were performed according to tertiles of eGDR to assess whether the associations differed across metabolic subgroups.

### Joint association of depression and VPA with CMM

**Figure 2:**
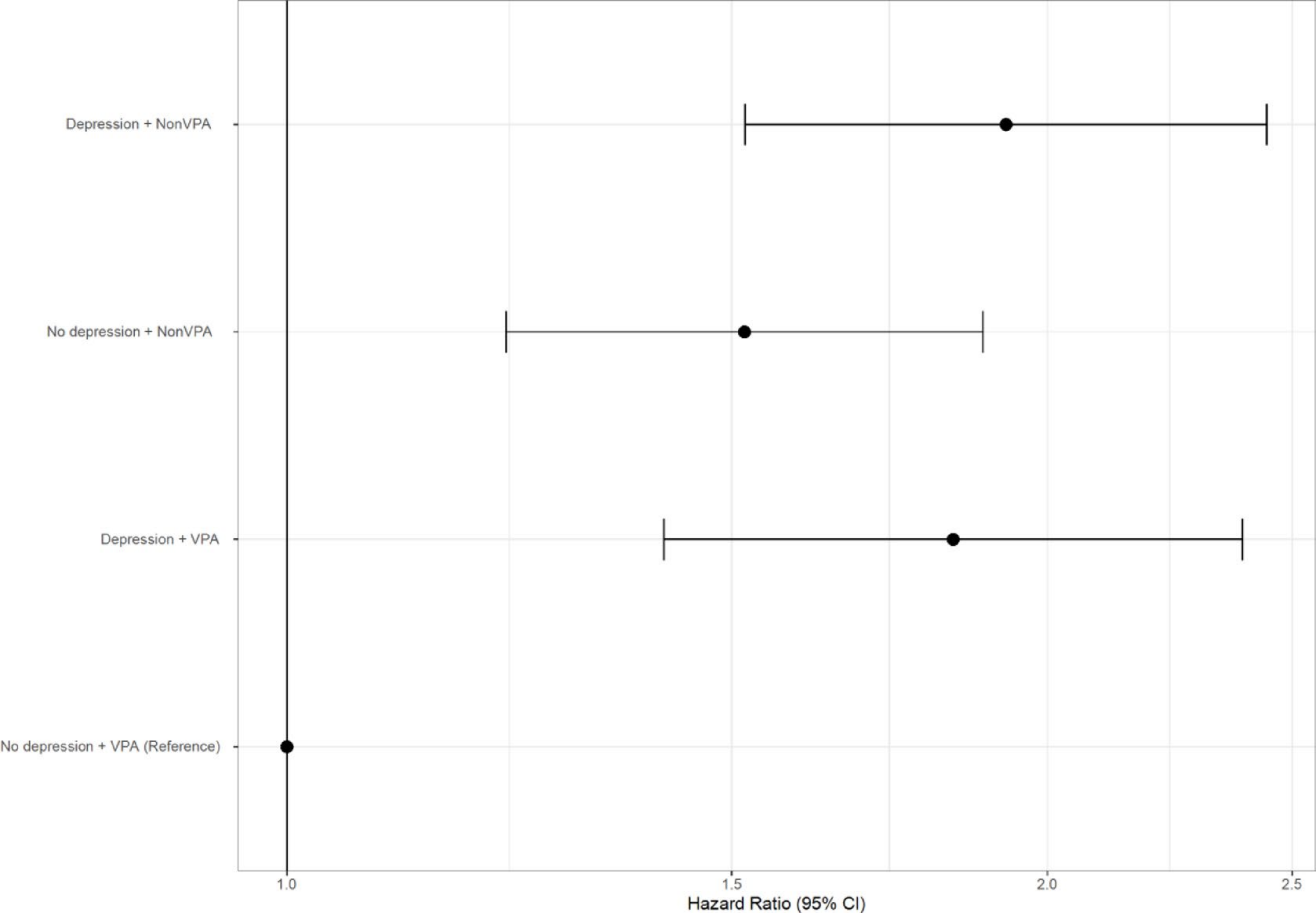
Joint associations of depression and VPA with incident CMM.

Figure 2 presents the joint associations of depression and VPA with CMM risk (Supplement Table S1). After accounting for differences in metabolic status by stratifying the Cox models according to eGDR tertiles, similar patterns of association were observed. Compared with participants without depression who engaged in VPA, those with depression who were physically active showed a moderately increased risk of CMM. Participants without depression but who did not engage in VPA also had an elevated risk. The highest risk was observed among individuals with both depression and no VPA (HR 1.93, 95% CI 1.52–2.44).

### Interaction analysis

To further evaluate whether the association between depression and incident CMM differed by physical activity status, an interaction term between depression and VPA was included in the fully adjusted Cox model. No evidence of interaction was observed (P for interaction = 0.073). However, a potential trend toward effect modification was noted, suggesting that the protective effect of VPA on CMM risk may be attenuated among individuals with depression.

### Interaction between joint exposure and eGDR

Interaction analyses were conducted to evaluate whether the association between the joint exposure of depression and VPA and CMM varied across eGDR strata. No evidence of interactions were observed between joint exposure and eGDR (all P for interaction > 0.05; Supplement Table S2). The associations between joint exposure and CMM remained generally consistent across different levels of metabolic status.

### Sensitivity analyses

**Figure 3:**
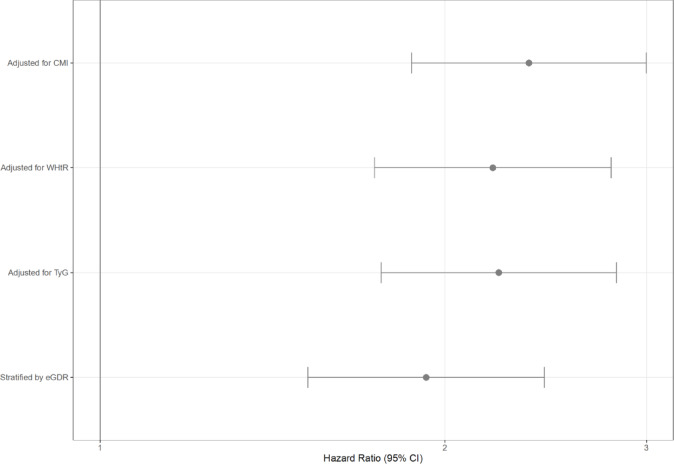
Sensitivity analyses of the association between depression combined with Non-VPA and CMM.

**Figure 4:**
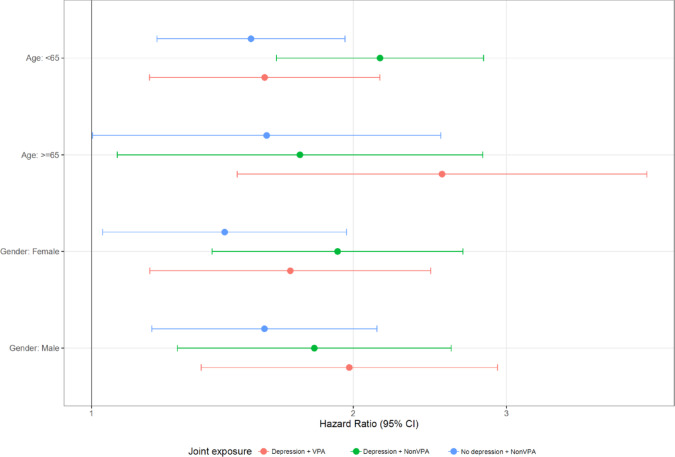
Forest plot of subgroup analysis by age and gender.

Sensitivity analyses (Supplementary Table S3) incorporating alternative metabolic indicators yielded consistent results. When the TyG index, WHtR, and CMI were included separately in fully adjusted models, the association between depression combined with physical inactivity and incident CMM remained robust (Fig. 3). These findings suggest that the observed associations were not driven by any single metabolic indicator and support the robustness of the main results. A linear trend was observed across increasing categories of the joint exposure of depression and physical activity (P for trend < 0.001). Subgroup analyses stratified by age and gender showed generally consistent associations, with no substantial heterogeneity observed across subgroups (Fig. 4).

### Sensitivity analysis excluding early incident cases

To further address potential reverse causality, we excluded participants who developed CMM during the first follow-up wave (2013), thereby minimizing the likelihood that preclinical or undiagnosed cardiometabolic conditions at baseline influenced depression status or physical activity levels. After this exclusion, the associations remained materially unchanged, indicating that reverse causation is unlikely to explain the observed relationships. In the fully adjusted model (Supplement Table S4), depression remained associated with an increased risk of CMM (HR 1.32, 95% CI 1.09–1.61), while VPA was associated with a reduced risk (HR 0.74, 95% CI 0.61–0.90).Similarly, in the joint exposure analysis (Supplement Table S5), participants with both depression and no VPA had the highest risk of CMM (HR 1.83, 95% CI 1.38–2.44), followed by those with depression who engaged in VPA (HR 1.61, 95% CI 1.18–2.20) and those without depression but no VPA (HR 1.53, 95% CI 1.19–1.98), compared with the reference group. Overall, the exclusion of early incident cases did not materially alter the observed associations, supporting the robustness of the findings.

## Discussion

In this prospective cohort study of middle-aged and older Chinese adults, we found that the joint presence of depression and low physical activity was associated with an increased risk of incident CMM. Compared with participants without depression who were physically active, those with coexisting depression and physical inactivity exhibited the highest risk. These associations remained robust after adjustment for a wide range of sociodemographic, behavioral, and health-related covariates, as well as across multiple sensitivity analyses incorporating alternative metabolic indicators.

Our findings extend previous evidence linking depression and physical inactivity to individual cardiometabolic diseases by demonstrating their combined impact on the progression toward multimorbidity. While earlier studies have shown that depression is associated with hypertension, diabetes, and cardiovascular disease, relatively few have examined its role in the accumulation of cardiometabolic conditions^38^. The present results suggest that depression may act as an upstream determinant that accelerates the clustering of cardiometabolic disorders, particularly when accompanied by insufficient physical activity.

To better interpret the observed association between the joint exposure of depression and physical inactivity and increased CMM risk, several biological and behavioral mechanisms may be involved. Depression has been linked to chronic activation of the hypothalamic–pituitary–adrenal axis, systemic inflammation, autonomic dysfunction, and insulin resistance, all of which contribute to cardiometabolic dysregulation^39–41^. These processes may be further exacerbated by physical inactivity, which impairs glucose utilization, promotes visceral adiposity, and worsens lipid metabolism. In contrast, VPA may exert protective effects through multiple pathways, including activation of AMP-activated protein kinase, enhanced fatty acid oxidation and glucose uptake, and improved myocardial energy metabolism^42^. Regular vigorous exercise has also been shown to reduce systemic inflammation and improve mitochondrial function, thereby mitigating cardiometabolic risk^43,44^. Together, these findings support the notion that depression and physical inactivity may act synergistically to promote cardiometabolic deterioration.

In the present study, we did not observe a statistical interaction between depression and VPA, although a marginal trend was noted. This suggests that while VPA is beneficial across exposure groups, its protective effect may be partially attenuated among individuals with depression. This pattern may reflect reduced adherence to physical activity, as well as persistent neuroendocrine and inflammatory dysregulation in depressed individuals^45,46^. Alternatively, the absence of evidence for interaction may reflect limited statistical power to detect modest effect modification.

To further address potential reverse causality, we conducted additional analyses excluding participants who developed CMM during the early follow-up period. The associations between depression, physical activity, and CMM remained largely unchanged. This suggests that the observed relationships are unlikely to be driven by preclinical or undiagnosed cardiometabolic conditions at baseline influencing depressive symptoms or physical activity levels. Importantly, the association between the joint exposure and CMM remained generally consistent across metabolic strata defined by eGDR. Although eGDR itself was strongly associated with CMM risk, we did not observe interaction effects between eGDR and the joint exposure. These findings suggest that metabolic status does not substantially modify the impact of depression and physical inactivity on CMM. In other words, the detrimental effects of this joint exposure appear to be broadly consistent across different metabolic risk profiles, highlighting that interventions targeting mental health and physical activity may be beneficial across a wide spectrum of baseline metabolic conditions^40,47^. Nevertheless, given that both depression and physical activity were assessed at baseline only, we cannot completely exclude the possibility of residual reverse causation or temporal changes in exposure status during follow-up.

From a public health perspective, these findings underscore the importance of integrated prevention strategies that simultaneously address mental health and lifestyle behaviors. Given the growing burden of multimorbidity in aging populations, interventions focusing solely on single diseases may be insufficient. Screening for depressive symptoms alongside promoting physical activity may represent a scalable and effective approach to reduce the risk of cardiometabolic disease clustering.

Several limitations should be acknowledged. First, depression and physical activity were assessed using self-reported measures, which may introduce misclassification. Second, both exposures were measured at baseline and may not capture changes over time, this limitation may have led to misclassification of the joint exposure categories, as participants may have changed their depression status or physical activity levels over time. Such misclassification is likely to be non-differential, which may have biased the associations toward the null and led to an underestimation of the true effects. Third, residual confounding, including the potential influence of medications, cannot be entirely excluded. Finally, the study population consisted of middle-aged and older Chinese adults, which may limit generalizability to other populations. Despite these limitations, the strengths of this study include its prospective design, nationally representative sample, repeated follow-up assessments, and comprehensive evaluation of metabolic status using multiple indicators.

## Conclusion

In conclusion, this study demonstrates that the coexistence of depression and physical inactivity is associated with a substantially increased risk of CMM among middle-aged and older adults. These findings support the integration of mental health promotion and physical activity interventions into public health strategies aimed at preventing CMM in aging populations.

## Supplementary Information

Below is the link to the electronic supplementary material.

Supplementary Material 1.

## Data Availability

The data that support the findings of this study are available from the China Health and Retirement Longitudinal Study (CHARLS). The datasets are publicly available upon registration at the CHARLS official website (http://charls.pku.edu.cn). The authors did not have special access privileges.
