# Supplementary material for: Joint association of depression and physical activity with incident cardiometabolic multimorbidity (CMM) among middle-aged and older adults in China
Source: Sci Rep. 2026 Apr 21;16:18417. doi: 10.1038/s41598-026-50014-2 (PMC13266066; doi:10.1038/s41598-026-50014-2)
Supplement: Supplementary file 1 — Supplementary Material 1. [file 41598_2026_50014_MOESM1_ESM.doc]

**Supplementary TableS1 joint associations of depression and VPA with CMM**

| term | HR | p.value | 95% lower | 95% high |
| --- | --- | --- | --- | --- |
| dep_vgact Depression + VPA | 1.836 | 0.00000704 | 1.41 | 2.389 |
| dep_vgact No depression + NonVPA | 1.518 | 0.000176 | 1.221 | 1.886 |
| dep_vgact Depression + NonVPA | 1.926 | 8.36E-08 | 1.518 | 2.443 |
| Age | 1.026 | 0.000000081 | 1.017 | 1.036 |
| Gender | 1.001 | 0.995 | 0.797 | 1.257 |
| Education | 1.083 | 0.0504 | 1 | 1.174 |
| Marry | 1.002 | 0.984 | 0.795 | 1.264 |
| IADL | 1.117 | 0.000877 | 1.047 | 1.192 |
| Smoke | 1.02 | 0.849 | 0.83 | 1.254 |
| Alcohol | 0.898 | 0.241 | 0.75 | 1.075 |
| Kidney disease | 1.751 | 0.0000338 | 1.345 | 2.28 |
| Respiratory function | 1 | 0.568 | 1 | 1.001 |
| Sleep time | 0.97 | 0.601 | 0.866 | 1.087 |
| Social activity | 1.055 | 0.189 | 0.974 | 1.143 |
| Fall_down | 1.123 | 0.208 | 0.937 | 1.345 |

**Supplementary TableS2 Interaction between joint exposure and eGDR**

| term | HR | p.value | 95% lower | 95% high |
| --- | --- | --- | --- | --- |
| Age | 1.0266033 | 4.84E-08 | 1.01704859 | 1.0362477 |
| Gender | 0.9871695 | 9.12E-01 | 0.78497881 | 1.2414394 |
| Education | 1.0879009 | 3.99E-02 | 1.00389876 | 1.1789321 |
| Marry | 1.0019325 | 9.87E-01 | 0.79394933 | 1.2643989 |
| IADL | 1.1197423 | 6.63E-04 | 1.0493272 | 1.1948826 |
| Smoke | 1.0246022 | 8.18E-01 | 0.83297711 | 1.2603103 |
| Alcohol | 0.8934364 | 2.22E-01 | 0.74545244 | 1.0707975 |
| Kidney disease | 1.7771559 | 2.21E-05 | 1.36422388 | 2.3150767 |
| Respiratory function | 1.0001953 | 5.69E-01 | 0.99952183 | 1.0008692 |
| Sleep time | 0.968798 | 5.85E-01 | 0.86455891 | 1.0856052 |
| Social activity | 1.0587951 | 1.61E-01 | 0.97745348 | 1.1469057 |
| Fall down | 1.1103185 | 2.57E-01 | 0.92635279 | 1.3308181 |
| Depression + VAP | 1.712688 | 2.73E-03 | 1.20534121 | 2.4335848 |
| Depression + NonVPA | 1.4685721 | 7.65E-03 | 1.10757818 | 1.947225 |
| Depression + NonVPA | 1.8630433 | 4.40E-05 | 1.38398054 | 2.5079329 |
| eGDR_groupMiddle | 0.2502225 | 5.47E-10 | 0.16233464 | 0.3856928 |
| eGDR_groupHigh | 0.1525934 | 1.73E-13 | 0.09330172 | 0.2495639 |
| **Depression+VAP: eGDR_group Middle** | 1.084215 | **8.00E-01** | 0.57942809 | 2.0287628 |
| **No depression + NonVPA: eGDR_groupMiddle** | 1.1600738 | **5.63E-01** | 0.70129583 | 1.9189779 |
| **Depression + NonVPA: eGDR_groupMiddle** | 1.2301803 | **4.48E-01** | 0.72029643 | 2.101001 |
| **Depression +VAP: eGDR_groupHigh** | 1.2663261 | **4.86E-01** | 0.6507538 | 2.4641911 |
| **No depression + NonVPA: eGDR_groupHigh** | 0.9450783 | **8.57E-01** | 0.51086442 | 1.7483562 |
| **Depression + NonVPA: eGDR_groupHigh** | 0.9032388 | **7.58E-01** | 0.47288139 | 1.7252537 |

**Supplementary TableS3 Sensitivity analyses**

| **Model** **Tyg** |  |  |  |  |
| --- | --- | --- | --- | --- |
|  | HR | p.value | 95% lower | 95% high |
| Age | 1.036212 | 1.10E-13 | 1.0266933 | 1.0458188 |
| Gender | 0.9659717 | 7.69E-01 | 0.7664939 | 1.2173632 |
| Education | 1.0825532 | 4.93E-02 | 1.0002545 | 1.1716233 |
| Marry | 1.1212349 | 3.39E-01 | 0.8866292 | 1.4179183 |
| IADL | 1.126481 | 4.25E-04 | 1.0544747 | 1.2034045 |
| Smoke | 0.9448896 | 5.95E-01 | 0.7664738 | 1.1648362 |
| Alcohol | 0.9979669 | 9.82E-01 | 0.8361528 | 1.1910957 |
| **VPA** | 0.7202492 | 6.39E-05 | 0.6136081 | 0.8454239 |
| Kidney disease | 1.6597266 | 1.61E-04 | 1.2768878 | 2.1573489 |
| Respiratory function | 1.0004543 | 1.99E-01 | 0.9997601 | 1.0011489 |
| Sleep time | 0.9786749 | 7.07E-01 | 0.874662 | 1.0950568 |
| Social activity | 1.0310765 | 4.51E-01 | 0.9521187 | 1.1165822 |
| Fall down | 1.1062894 | 2.75E-01 | 0.9225082 | 1.3266834 |
| **Depression** | 1.4981265 | 5.52E-07 | 1.2802955 | 1.7530194 |
| Tyg | 1.7732924 | 7.82E-28 | 1.6063982 | 1.9575257 |
| **Model WHtR** |  |  |  |  |
| Age | 1.0297229 | 8.29004E-10 | 1.0202441 | 1.0392898 |
| Gender | 1.0791894 | 0.5251107 | 0.8529112 | 1.3654995 |
| Education | 1.091291 | 0.0312341 | 1.0079243 | 1.181553 |
| Marry | 1.0618699 | 0.6145715 | 0.840384 | 1.3417293 |
| IADL | 1.1228842 | 0.000631803 | 1.050852 | 1.199854 |
| Smoke | 1.0652977 | 0.5479623 | 0.8664726 | 1.3097462 |
| Alcohol | 1.0214711 | 0.8154768 | 0.8543669 | 1.2212589 |
| **VPA** | 0.7094541 | 2.75723E-05 | 0.6047071 | 0.8323452 |
| Kidney disease | 1.6399531 | 0.000230573 | 1.2614363 | 2.1320505 |
| Respiratory function | 1.0003749 | 0.2880035 | 0.9996826 | 1.0010677 |
| Sleep time | 0.9721275 | 0.625576 | 0.8676699 | 1.0891607 |
| Social activity | 1.0573322 | 0.1746025 | 0.9755147 | 1.1460119 |
| Fall down | 1.088415 | 0.3613625 | 0.9072141 | 1.3058078 |
| **Depression** | 1.47983 | 1.20884E-06 | 1.2645187 | 1.7318027 |
| WHtR | 274.6070845 | 1.42062E-22 | 92.1035358 | 818.7421929 |
| **Model CMI** |  |  |  |  |
| Age | 1.0358713 | 1.19112E-13 | 1.0264289 | 1.0454005 |
| Gender | 0.8756789 | 0.2599979 | 0.694927 | 1.1034449 |
| Education | 1.0785912 | 0.05985947 | 0.9968559 | 1.1670283 |
| Marry | 1.1719011 | 0.1887891 | 0.9248598 | 1.4849302 |
| IADL | 1.1357018 | 0.000169353 | 1.0630644 | 1.2133024 |
| Smoke | 0.9803597 | 0.8519758 | 0.7957736 | 1.2077622 |
| Alcohol | 1.0251954 | 0.7826543 | 0.8588807 | 1.2237156 |
| **VPA** | 0.6947119 | 9.59904E-06 | 0.5917099 | 0.8156439 |
| Kidney disease | 1.7004188 | 7.57403E-05 | 1.3086148 | 2.2095304 |
| Respiratory function | 1.0004622 | 0.1916228 | 0.9997679 | 1.0011569 |
| Sleep time | 0.9970103 | 0.9586618 | 0.8901597 | 1.1166867 |
| Social activity | 1.0404224 | 0.3282032 | 0.9609019 | 1.1265238 |
| Fall down | 1.1101403 | 0.2590952 | 0.9257692 | 1.3312298 |
| **Depression** | 1.4749557 | 1.37234E-06 | 1.2609813 | 1.7252392 |
| CMI | 1.0747851 | 3.72806E-13 | 1.0544002 | 1.0955641 |

**Supplement Table S4 Sensitivity analysis excluding early incident cases(fully adjusted model)**

| term | HR | p.value | 95% lower | 95% high |
| --- | --- | --- | --- | --- |
| Age | 1.0289 | <0.001 | 1.0173 | 1.0406 |
| Gender | 0.8218 | 0.183 | 0.6153 | 1.0975 |
| Education | 1.0962 | 0.065 | 0.9943 | 1.2085 |
| Marry | 1.0425 | 0.779 | 0.7793 | 1.3946 |
| IADL | 1.1584 | <0.001 | 1.0652 | 1.2597 |
| Smoke | 1.0793 | 0.566 | 0.8311 | 1.4016 |
| Alcohol | 1.1491 | 0.211 | 0.9240 | 1.4290 |
| **VPA** | 0.7421 | 0.003 | 0.6116 | 0.9005 |
| Kidney disease | 1.3974 | 0.063 | 0.9817 | 1.9891 |
| Respiratory function | 0.9998 | 0.695 | 0.9990 | 1.0007 |
| Sleep time | 1.0449 | 0.536 | 0.9088 | 1.2015 |
| Social activity | 0.9665 | 0.522 | 0.8705 | 1.0730 |
| Fall down | 1.0909 | 0.457 | 0.8669 | 1.3728 |
| **Depression** | 1.3230 | 0.005 | 1.0892 | 1.6069 |

**Supplement TableS5 Sensitivity analysis excluding early incident cases (joint exposure analysis)**

| term | HR | p.value | 95% lower | 95% high |
| --- | --- | --- | --- | --- |
| dep_vgact No depression + VPA |  |  |  |  |
| dep_vgact Depression + VPA | 1.6119 | 0.0029 | 1.1787 | 2.2043 |
| dep_vgact No depression + NonVPA | 1.5338 | 0.0011 | 1.1883 | 1.9797 |
| dep_vgact Depression + NonVPA | 1.8318 | <0.001 | 1.3759 | 2.4386 |
| Age | 1.0292 | <0.001 | 1.0175 | 1.0409 |
| Gender | 0.8151 | 0.165 | 0.6105 | 1.0884 |
| Education | 1.0963 | 0.065 | 0.9943 | 1.2086 |
| Marry | 1.0384 | 0.799 | 0.7762 | 1.3890 |
| IADL | 1.1641 | <0.001 | 1.0701 | 1.2662 |
| Smoke | 1.0881 | 0.526 | 0.8377 | 1.4133 |
| Alcohol | 1.1501 | 0.208 | 0.9250 | 1.4299 |
| Kidney disease | 1.3857 | 0.070 | 0.9732 | 1.9732 |
| Respiratory function | 1.0000 | 0.749 | 0.9990 | 1.0007 |
| Sleep time | 1.0442 | 0.543 | 0.9081 | 1.2006 |
| Social activity | 0.9659 | 0.515 | 0.8698 | 1.0726 |
| Fall down | 1.0878 | 0.472 | 0.8644 | 1.3690 |
